# Multizonal outer retinopathy and retinal pigment epitheliopathy (MORR) with a chronologically divergent presentation- a case report

**DOI:** 10.1186/s12348-025-00519-0

**Published:** 2025-07-22

**Authors:** Blerta Lang, Karl Boden, Boris V. Stanzel, Ulrich Prothmann, Peter Szurman

**Affiliations:** 1 Eye Clinic Sulzbach, Knappschaft Kliniken Saar GmbH, An der Klinik 10, Sulzbach, D-66280 Germany; 2 Klaus Heimann Eye Research Institute, An der Klinik 10, Sulzbach/Saar, D-66280 Germany; 3 Püttlingen Rheumatology Clinic, Knappschaft Kliniken Saar GmbH, In der Humes 35, Püttlingen, D-66346 Germany; 4 Department of Ophthalmology, University of Bonn, Bonn, Germany

**Keywords:** Multizonal outer retinopathy and retinal pigment epitheliopathy, Acute zonal occult outer retinopathy, Course, Multimodal imaging

## Abstract

**Purpose:**

Acute zonal occult outer retinopathy (AZOOR) is a rare inflammatory disease of the outer retina, often presented with subtle early findings. A specific subtype, termed Multizonal Outer Retinopathy and Retinal Pigment Epitheliopathy (MORR), is characterized by distinct progression pattern (Ramtohul et al. Retina 43:1890–1903, 2023) in multiple zones of the outer retina and retinal pigment epithelium. This case report aims to illustrate the chronologically divergent presentation, a phase-shifting disease progression and the complex clinical course of MORR, and to discuss the diagnostic challenges posed by its phase‑shifted timeline as well as potential therapeutic options.

**Methods:**

A case of a 52-year-old female patient with initially unilateral, inactive posterior uveitis was retrospectively analyzed. Over thirteen years, progressive functional impairment developed in the fellow eye. Findings were assessed using multimodal imaging (Optical Coherence Tomography [OCT], Fundus Autofluorescence [FAF], Fluorescein and Indocyanine Green Angiography [FFA/ICGA]) and electrophysiological examinations (multifocal ERG, full-field ERG, EOG, VEP). Additional rheumatologic and neurologic assessments were conducted, and an infectious workup was performed.

**Results:**

The patient demonstrated chronologically divergent bilateral involvement with extensive damage to the retinal pigment epithelium (RPE) and photoreceptors. Fundus autofluorescence revealed a tri- to multizonal pattern in the better eye, while the fellow eye already exhibited diffuse atrophic areas devoid of any autofluorescence. Electrophysiological, the better eye showed selectively prolonged latencies on multifocal electroretinography (multifocal ERG) but preserved amplitudes, whereas the more severely affected eye displayed substantial functional loss. Despite various therapeutic interventions, including high-dose corticosteroids and immunosuppressive agents, progressive visual impairment ensued, driven by increasing macular involvement.

**Conclusions:**

This case highlights the marked heterogeneity and diagnostic complexities of Multizonal Outer Retinopathy and Retinal Pigment Epitheliopathy (MORR), a newly recognized progressive variant of Acute Zonal Occult Outer Retinopathy (AZOOR). The disease course can present with chronologically divergent manifestations in both eyes. While initial stages may exhibit only subtle funduscopic changes, structural and functional deficits can progress rapidly, episodically and in a phase-shifted manner. Multimodal imaging is essential to delineate the disease trajectory and to distinguish MORR from diseases affecting outer retina. Currently, no definitive treatment is available; although immunomodulatory therapies may stabilize the condition in certain cases, their efficacy remains inconsistent. Consequently, early low-vision management and close interdisciplinary collaboration are of particular importance.

## Introduction

Acute zonal occult outer retinopathy (AZOOR) and its variant, Multizonal Outer Retinopathy and Retinal Pigment Epitheliopathy (MORR), are rare inflammatory disorders primarily affecting the outer retina and retinal pigment epithelium (RPE). They pose substantial diagnostic and therapeutic challenges due to their heterogeneous presentations and frequent subtle early findings.

We report a chronologically divergent, bilateral MORR presentation in a 52-year-old woman initially diagnosed with unilateral, inactive posterior uveitis. Over eight years, she developed progressive functional impairment in the fellow eye. Multimodal imaging and electrophysiological testing proved crucial for accurate diagnosis and monitoring.

MORR is characterized by multiple outer retinal and RPE lesions, often with more pronounced RPE involvement than classic AZOOR. Its etiology remains uncertain, with viral infections, autoimmune mechanisms, and systemic associations among the potential contributing factors.

By highlighting this complex clinical course and the utility of advanced imaging in diagnosis, we aim to increase awareness of MORR, emphasize the need for careful differential diagnosis within the AZOOR spectrum, and encourage further research into this challenging entity.

## Case report

A 52-year-old, otherwise healthy woman presented with a previously diagnosed, idiopathic, left-sided, inactive posterior uveitis. She reported occasional photopsia in the right eye, while the left eye had already developed advanced visual impairment starting at age 32, following an unclear retinitis during a one-month stay in Los Angeles. At that time, she experienced nonspecific malaise, headaches, and gastrointestinal discomfort initially deemed flu-like. She had a known history of migraine, with no evidence of mononucleosis, herpes dermatitis, weight loss, night sweats, or insect bites. She denied any recent vaccinations, tobacco use, or malignancy. Shortly before turning thirty-two, she underwent estrogen-based hormonal therapy for fertility. The patient also reported allergies to penicillin, novalgina, diclofenac, and latex.

At presentation, the best-corrected visual acuity (BCVA) measured 20/20 OD (+ 0.25/−0.25/A 11) and 20/200 OS (+ 0.25/−0.75/A 116). Intraocular pressure was 14 mmHg in both eyes, and the anterior segment showed no signs of inflammation.

Funduscopic examination of the right eye revealed a vital, sharply defined optic disc, a dry macula, and peripheral degenerations of uncertain classification. The left eye showed a slightly pale optic disc, diffuse chorioretinal atrophy with pigment shifts in the macula, and peripheral chorioretinal scars.

**Fig. 1:**
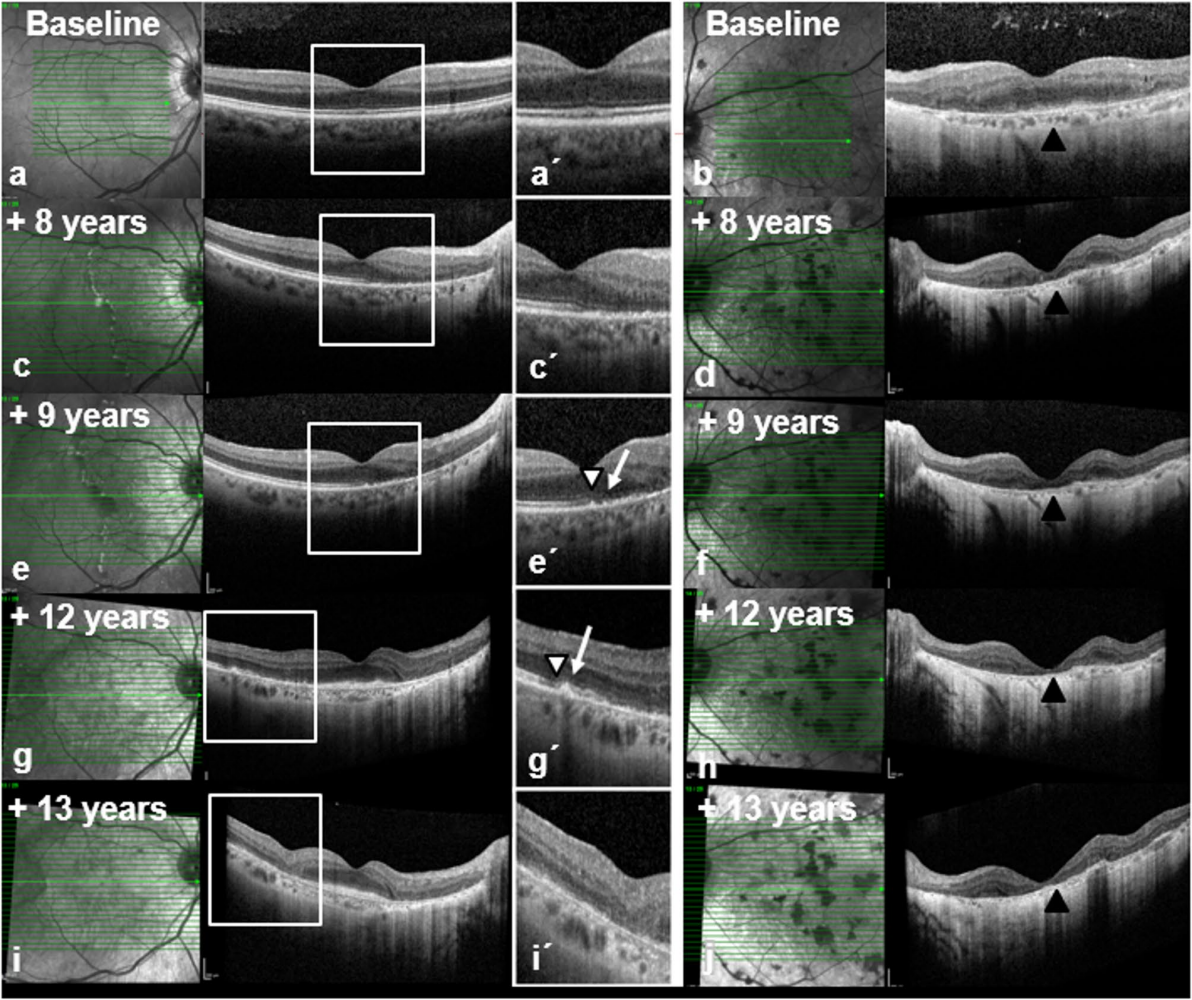
Fundus autofluorescence (FAF; Spectralis, Heidelberg Engineering, Heidelberg: a-c, a´-c, Clarus 500, Zeiss, Oberkochen, Germany). Both eyes over time (Upper row, a-d, right eye, lower row, a’-d’, left eye) The figure shows a peripapillary lesion with a trizonal autofluorescent pattern in the right eye: linear to spotty flaring hyperautofluorescent border between MORR lesion and normal retina (Zone I), speckled hyperautofluorescence within the MORR lesion (Zone II) and hypoautofluorescent area corresponding to RPE to choroidal atrophy (Zone III), with a progressive course over the years (upper row). At the 12th year of presentation, centripetal spread from inferior with mild speckled hyperfluorescent border is seen (white triangle). At the 13th year of presentation, the centripetal multizonal spread formed a subtotal circular pattern with confluent areas and showed speckled hyperautofluorescence at the margin at the transition to normal retina. The periphery showed confluent hypoautofluorescence corresponding to the extensive RPE atrophy (white triangle). The left eye shows similar absent autofluorescence over the years due to central retinal to choroidal atrophy. Individual patchy hypo- to isoautofluorescent lesions correspond to the inhomogeneously distributed pigment clumping (lower row)

Optical coherence tomography (OCT) of the right eye demonstrated only a minimally blurred foveal ellipsoid zone without disruption of the outer retina (Fig. 1a, a′, “Baseline”). In the left eye (Fig. 1b, “Baseline”), OCT revealed extensive para- to perifoveal atrophy of the outer retinal layers, irregular outer layering from sub- to perifoveal, and mild macular thickening.

### Course, diagnosis and treatment

At age 60, eight years later, the patient returned with progressive visual impairment, flickering, temporal field restriction, and photopsia in the right (better) eye. In the left eye, dark/gray vision remained stable. She reported no additional systemic infections but had HLA-B27-positive spondylarthritis and stress-related complaints. Best-corrected visual acuity dropped to 20/30 OD and light perception OS.

**Fig. 2:**
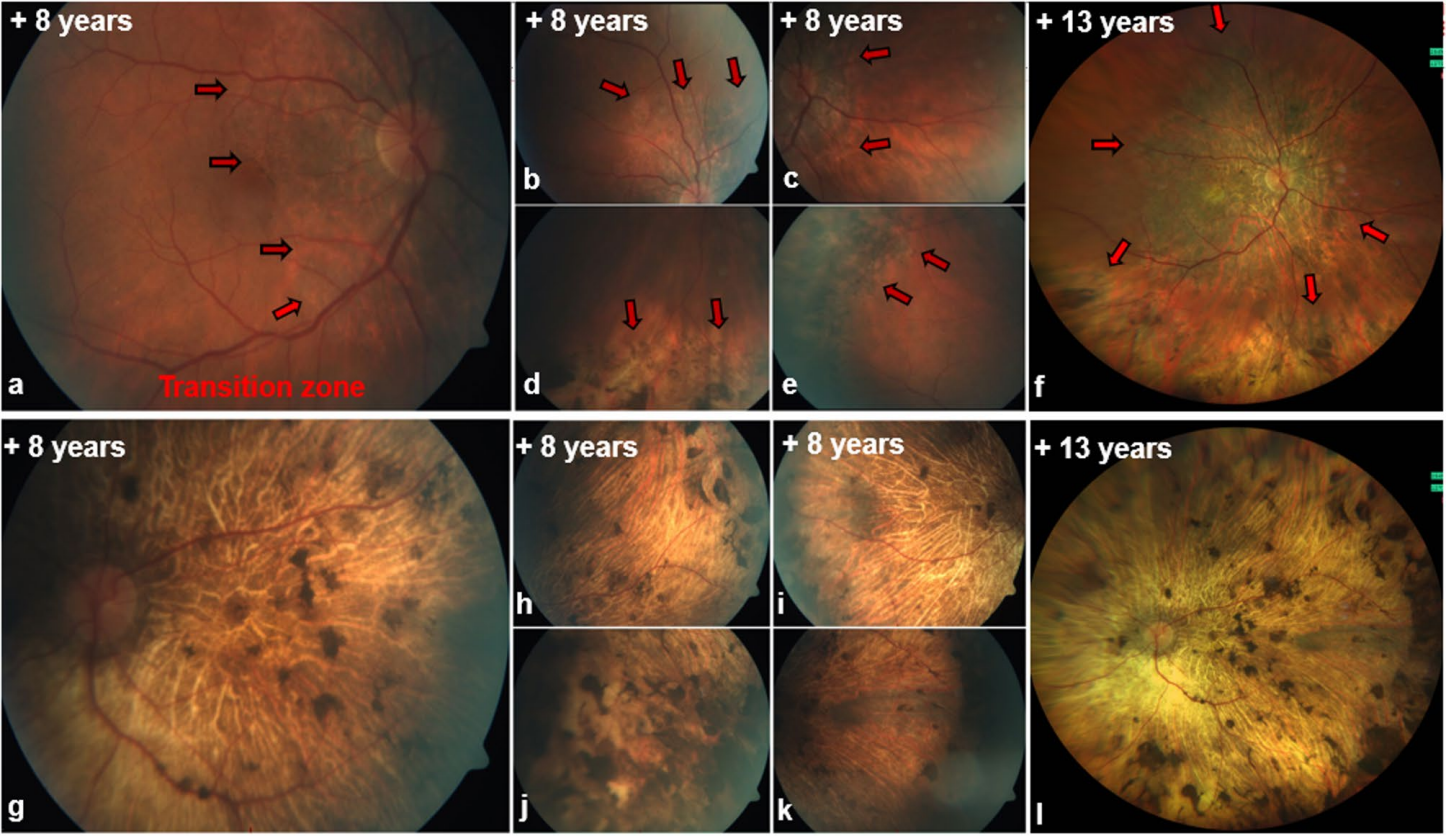
Correlation of FFA, ICGA and SD-OCT for photoreceptor, RPE and choriocapillaris atrophy zones The white arrows show three border transitions in the right eye (a, c): (I) from normal anatomical outer retinal structures and choriocapillaris to (II) RPE and photoreceptor degeneration/remodeling with reticular-like drusenoid deposits in OCT and corresponding hypo- or hyperfluorescent appearance in fluorescence angiography depending on their position in relation to the pigment layer of the retina and the amount of lipids and proteins they contain, missing drusen fluorescence in RPE atrophy. Subsequently (III) complete RPE loss and limiting to absent visualization of the choriocapillaris with decrease of peripapillary choroidal thickness in OCT, corresponding window defect and simultaneously hypofluorescent in FFA, as well as peripapillary vessel density decrease (yellow arrows) of the choroid in ICG

Funduscopic examination of the right eye (Fig. 2a-e) revealed a sharply defined optic disc, chorioatrophy from peripapillary to nasal parafoveal, dry macula with pigment shifts, and chorioatrophic scars. A distinct transition zone between atrophic and normal retina was evident (red arrowheads). By contrast, the left eye (Fig. 2g–k) exhibited a pale optic disc, diffuse atrophy, and patchy, irregular pigment clumping.

**Fig. 3:**
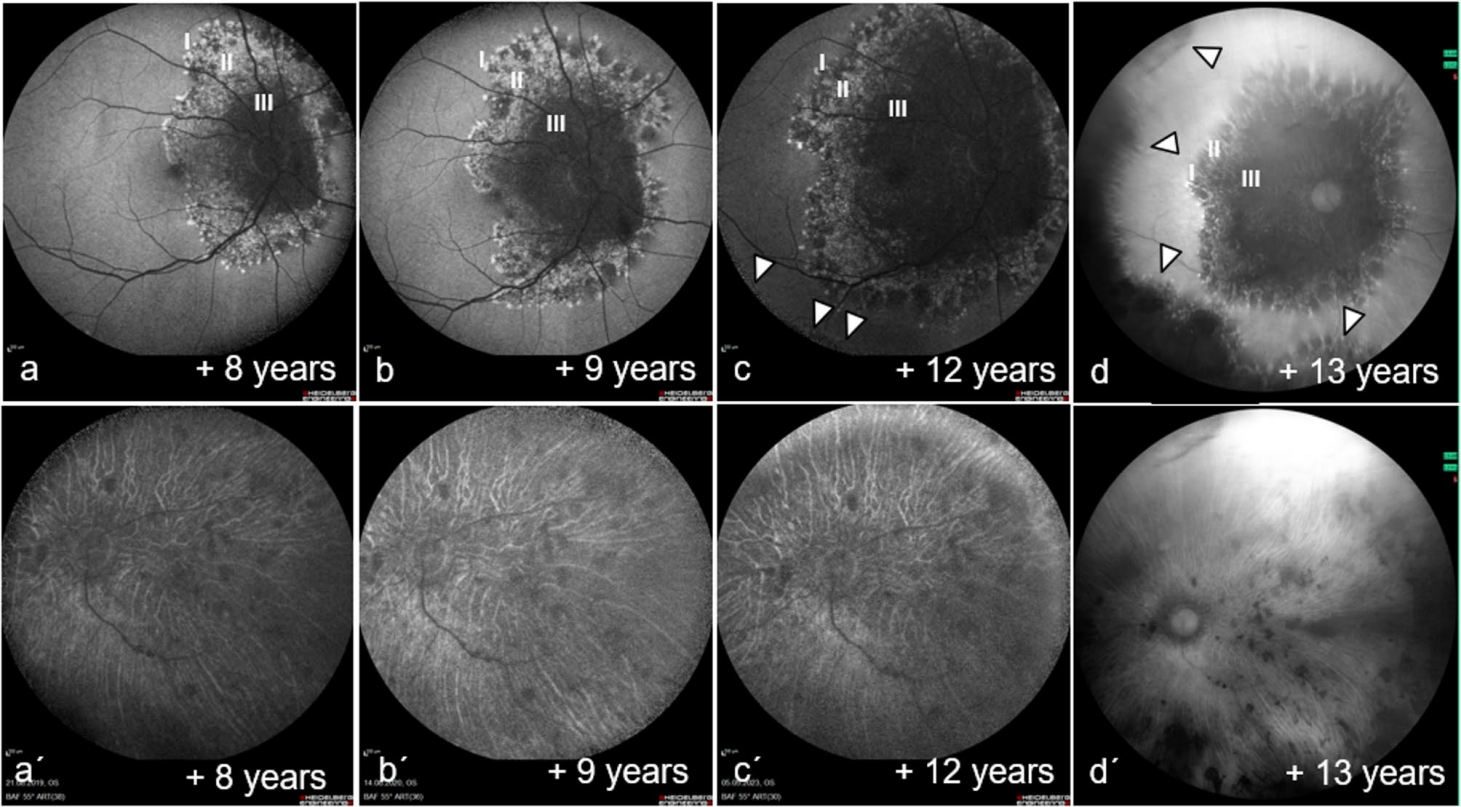
∆: SD-OCT progression over 13 years

Blue fundus autofluorescence (BAF, Spectralis^®^, Heidelberg Engineering, Germany) of the right eye (Fig. 3a) showed a central trizonal autofluorescent pattern: A linear to punctually flaring hyperautofluorescent border between the lesion and normal retina (Zone I), a speckled hyperautofluorescence within the lesion (Zone II), and a hypoautofluorescent area corresponding to RPE and choroidal atrophy (Zone III). The left eye (Fig. 3a’) lacked any apparent autofluorescence due to central retinal and choroidal atrophy.

On OCT, the right eye **(**Fig. 1c, c’, enlarged macular area) showed a nasal outer retinal thinning and ellipsoid zone (EZ) disruption. The RPE-Bruch’s membrane complex appeared fragmented, with hyperreflective spots. The external limiting membrane (ELM) can be traced to perifoveal nasal. The outer nuclear layer (ONL) appeared shortened, due to layer shifting and the partly apparent elongation of the outer plexiform layer (OPL). By contrast, the left eye (Fig. 1d) displayed complete outerretinal atrophy and markedly reduced choroidal thickness.

**Fig. 4:**
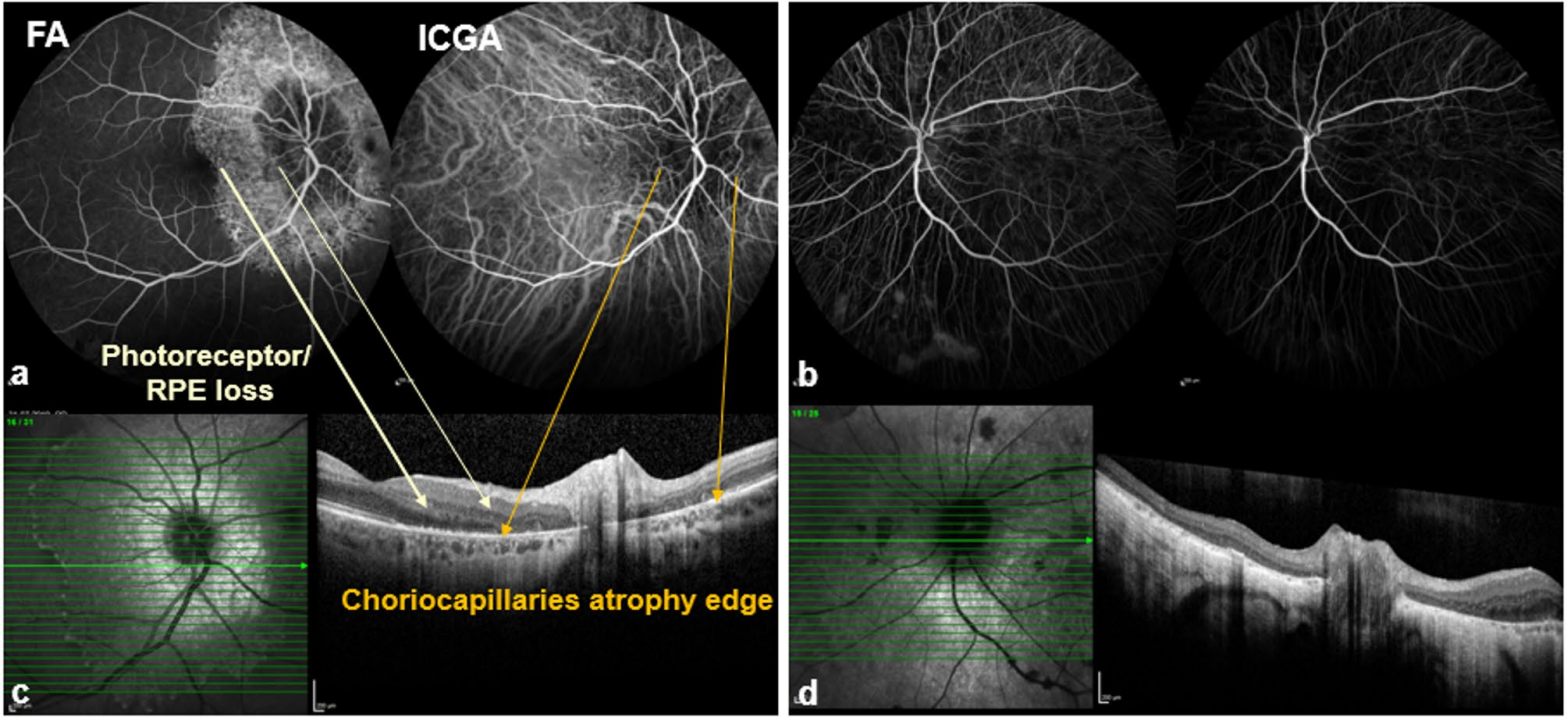
∆: Fundus photographs demonstrating progressive atrophy and pigment changes in both eyes over time

Fluorescein angiography (FFA) of the right eye (Fig. 4a) showed delayed vessel filling (armretina time 36 s, measured at the right eye), a peripapillary hypofluorescence and subsequent diffuse hyperfluorescence; indocyaninegreen angiography (ICGA) revealed reduced choroidal vessel density. In the left eye (Fig. 4b), severe atrophy abolished retinal fluorescence and produced marked choroidal hypofluorescence.

The corresponding peripapillary OCT of the right eye (Fig. 4c) showed three transition zones: (I) From normal outer retinal structure and intact choriocapillaris, (II) to RPE and photoreceptor degeneration or remodeling with reticular-like drusenoid deposits, (III) to complete RPE loss with only limited or absent visualization of the choriocapillaris and reduced peripapillary choroidal thickness.

Peripapillary OCT (Fig. 4d) confirmed in the left eye the extensive damage to the outer retinal layers and an almost complete loss of the peripapillary choroidal layer.

Electrophysiological testing: In the right eye, multifocal ERG revealed intact central amplitudes but prolonged implicit time. By contrast, the left eye showed markedly reduced amplitudes, indicating severe global dysfunction. Full-field ERG was age-appropriate in the right eye but severely diminished in the left, with reduced amplitudes and prolonged latencies under both scotopic and photopic conditions. EOG yielded an Arden quotient of 2.2 (right) and 1.4 (left). Pattern VEP latencies were normal in both eyes; however, left-eye amplitudes were notably reduced.

With the abovementioned taken together, the findings thus pointed further towards the diagnosis of AZOOR.

Systemic evaluation ruled out significant viral/infectious causes (e.g., HIV, VZV, CMV, EBV).

Systemic comorbidities included mild HLA-B27-positive spondylarthritis, degenerative skeletal changes with right-convex scoliosis, isolated enthesopathies including diabetes mellitus and substituted hypothyroidism. Neurological co-assessment revealed no pathological findings.

To exclude paraneoplasia, comprehensive laboratory tests were performed, including hormonal assessments (FSH, LH, growth hormone, prolactin, IGF-1, and androstenedione), all of which were normal. Cancer-associated retinopathy was effectively ruled out by the absence of enolase, recoverin, and Hu-antibodies (anti-Hu/ANNA-1). MRI of the upper abdomen and pelvis showed no tumor processes. Dermatological examination revealed only seborrheic keratosis and compound nevi, with no suspicious lesions. Apart from asymptomatic reflux esophagitis, gastro- and colonoscopy were likewise unremarkable. A bone scan indicated no metastatic lesions, showing only focal activity consistent with degenerative joint and spinal changes.

Given macular involvement, systemic prednisolone (100 mg/day for 5 days, taper over 12 weeks) was administered, followed by attempts at immunomodulation (methotrexate, then adalimumab) in cooperation with the rheumatology clinic in Püttlingen, but adverse effects led to discontinuation. During immunomodulation therapy, the patient experienced severe hair loss, persistent gastrointestinal upset, headaches, pronounced fatigue with myalgia, a constant flulike malaise, and newonset depressive symptoms that led to social withdrawal.

To enhance her visual support, the patient received an electronic handheld magnifier (“pebble” from Enhanced Vision), a 7× illuminated handheld magnifier (Mobilux, Eschenbach), magnification software (ZoomText), and information on smartphone apps and other daily living aids (Marland catalog). The 3.5× Mobilux handheld magnifier with a stand proved the most comfortable solution.

Further follow-ups (+ 9, + 12, +13 years) showed continued progression on OCT (OD: Fig. 1e, e’, 1 g, g’, 1 i, i´,/OS: Figure 1f, h and j), and autofluorescence (OD: Fig. 3b-d/OS: b´- d´), predominantly in the right eye, with residual atrophy in the left. Despite therapy, vision deteriorated to light perception or worse in both eyes.

From these extensive, multizonal findings, MORR was diagnosed, representing a variant of AZOOR.

## Discussion

AZOOR, first described by Gass in 1992, is a rare retinal disease marked by abrupt photoreceptor dysfunction despite minimal fundus changes in initial stages [1–3]. A variant termed MORR exhibits stronger RPE involvement than classic AZOOR [4]. Clinically, AZOOR often presents unilaterally, but can later affect the fellow eye. MORR, however, typically manifests bilaterally with scotomas and photopsia, yet may also progress in an episodic and asymmetric pattern [3, 4]. Both conditions primarily affect young to middle-aged women; in AZOOR, the mean onset is 20–40 years [5], potentially influenced by hormonal or genetic factors [6]. MORR likewise has been reported mostly in women aged 39–71 [3].

Although the pathogenesis remains unclear, evidence supports viral triggers (EBV, HSV, HZV, CMV) and autoimmune mechanisms [7–10]. Antiretinal antibodies (e.g., CRALBP, recoverin, α-enolase, S-antigen) have been found in AZOOR [7–10], but also occur in other diseases, complicating diagnosis. Genetic predisposition for AZOOR appears absent [11]. Additional associations include vaccinations, tick bites, migraine, and fetomaternal cell migration [9, 12]. Systemic autoimmune conditions, especially thyroid disorders—have been reported in both AZOOR and MORR [3], suggesting shared immunologic pathways.

MORR and acute annular outer retinopathy (AAOR) are now regarded as special AZOOR variants, based on evolving multimodal imaging [13, 14]. As part of the “AZOOR complex,” these entities often mimic other inflammatory conditions such as multiple evanescent white dot syndrome (MEWDS), punctate inner choroidopathy (PIC), acute macular neuroretinopathy (AMN), idiopathic blind spot enlargement(IBSE), acute idiopathic maculopathy (AIM), and autoimmune retinopathies [15, 16].

Funduscopic findings in AZOOR may appear normal initially, followed by pigment shifts and chorioretinal atrophy, often in zonal patterns. MORR is distinguished by early, multizonal RPE changes with hyper-/hypoautofluorescent lesions on FAF [3, 17]. On angiography, AZOOR typically shows discrete RPE “window defects,” whereas MORR can exhibit more complex, widespread fluorescence abnormalities [3]. In indocyanine green angiography (ICGA), AZOOR might have normal choriocapillaris perfusion or display focal hypofluorescence [18], while MORR reveals broader choroidal involvement [3]. OCT in AZOOR frequently demonstrates selective outer retinal atrophy, whereas MORR shows extensive RPE disruption and choroidal thinning, sparing the inner retina [3, 17].

Both conditions can show regional amplitude reductions on multifocal ERG, reflecting focal photoreceptor damage [6, 19]. In AZOOR, full-field ERG is often normal unless the disease becomes more diffuse [20]. MORR may present broader amplitude loss across multiple retinal zones. Pattern VEP changes are usually minimal unless the central macula is severely involved.

No standardized therapy exists for AZOOR or MORR. Spontaneous remission can occur in AZOOR but is rarer in MORR [19]. Treatment options—corticosteroids, immunomodulators (e.g., methotrexate, adalimumab), antivirals—have produced inconsistent outcomes [5, 21]. A case series of 10 MORR patients revealed that they received systemic or intravitreal therapies without uniform benefit [3]. However, multiple intravitreal injections of longacting corticosteroid implants achieved temporary stabilization and partial lesion regression [4]. Intravitreal steroids have likewise been shown to stabilize disease in selected AZOOR cases [22]. Our patient’s disease likewise progressed despite systemic steroids and biologics, and intravitreal corticosteroid therapy was declined, underscoring the limited efficacy of current regimens. Intravitreal corticosteroid implant was denied. Both AZOOR and MORR exhibit highly variable, potentially relapsing courses, with final vision dictated by the extent of outer retinal involvement [3, 21].

A comparative table (Table 1) summarizes the main distinctions between MORR and AZOOR, based on the literature discussed. It emphasizes the critical role of multimodal imaging in accurate diagnosis and clinical management of these conditions.

**Table 1:** Comparative table highlighting key distinctions between MORR and AZOOR from the discussed literature

Hallmark	MORR	AZOOR
Primary Involvement	Outer retina and retinal pigment epithelium (RPE) in multiple zones	Primarily the outer retina; early fundus often unremarkable
RPE Changes	Pronounced, with extensive atrophy and pigment disruption from the outset	Mild or absent at initial stages; RPE involvement typically emerges later
Fundus Autofluorescence (FAF)	Multizonal hyper-/hypoautofluorescent patterns; reflect larger RPE damage	May show zonal or patchy FAF changes; initially subtle
Onset & Laterality	Often bilateral from the start or becomes bilateral in a staggered, progressive manner	Commonly unilateral at first, can affect the fellow eye later; can also appear asymmetric
Demographics	Mostly females, typically 39–71 years	Mostly females, typically 20–40 years (often myopic)
Electrophysiology (ERG, multifocal ERG)	Diffuse or multizonal amplitude reductions; more extensive photoreceptor dysfunction	Focal or zonal amplitude reductions can be normal until later stages
Imaging (e.g., OCT, ICGA)	Shows widespread outer retinal disruption and choroidal thinning (ICGA often more pronounced)	Selective outer retinal atrophy; ICGA can be normal or show focal hypofluorescence
Prognosis & Therapy	Tends to be more progressive; no standardized treatment; immunosuppression often attempted Consider intravitreal steroids	Variable course: may stabilize spontaneously, others progress; immunomodulatory therapy tried, Intravitreal steroids can achieve disease stability
Distinctive Feature	Early and pronounced RPE involvement (explaining more severe, multizonal changes)	Primarily photoreceptor-focused damage, often sparing the RPE in initial stages

Overall, this case highlights the diagnostic and therapeutic challenges posed by MORR as an AZOOR variant. Comprehensive imaging, electrophysiological assessment, and exclusion of infectious or autoimmune differentials remain paramount. Further research is needed to clarify pathogenesis and develop more effective treatments.

## Conclusion

This case underscores the diagnostic complexities and management challenges of MORR, a variant of AZOOR. Both conditions pose diagnostic and therapeutic challenges, necessitating multimodal imaging and a high index of suspicion for accurate diagnosis. The chronologically divergent, bilateral involvement, progressive disease course, and limited treatment efficacy highlight the need for vigilant monitoring and interdisciplinary collaboration. Further research is essential to clarify the pathogenesis of these conditions and to develop more effective therapeutic approaches.

## Data Availability

No datasets were generated or analysed during the current study.

## Abbreviations

AIM: Acute idiopathic maculopathy
AMN: Acute macular neuroretinopathy
AZOOR: Acute Zonal Occult Outer Retinopathy
BAF: Blue fundus autofluorescence
BCVA: Best-corrected visual acuity
CMV: Cytomegalovirus
EBV: Epstein-Barr vi
ELM: External limiting membrane
EOG: Electrooculography
EZ: Ellipsoid zone
FAF: Fundus autofluorescence
FFA: Fluorescein angiography
Full-field ERG: Full-field electroretinography
HIV: Human immunodeficiency virus
IBSE: Idiopathic blind spot enlargement
ICG: Indocyanine green angiography
ISCEV: International Society for Clinical Electrophysiology of Vision
MEWDS: Multiple evanescent white dot syndrome
Multifocal ERG: Multifocal electroretinography
MORR: Multizonal Outer Retinopathy and Retinal Pigment Epitheliopathy
OCT: Optical coherence tomography
ONL: Outer nuclear layer
OPL: Outer plexiform layer
PIC: Punctate inner choroidopathy
RPE: Retinal pigment epithelium
VEP: Visual evoked potentials
VZV: Varicella-zoster virus
